# Molecular Epidemiology of Clade 1 Influenza A Viruses (H5N1), Southern Indochina Peninsula, 2004–2007

**DOI:** 10.3201/eid1510.090115

**Published:** 2009-10

**Authors:** Philippe Buchy, Mathieu Fourment, Sek Mardy, San Sorn, Davun Holl, Sowath Ly, Sirenda Vong, Vincent Enouf, J.S. Malik Peiris, Silvie van der Werf

**Affiliations:** Institut Pasteur, Phnom Penh, Cambodia (P. Buchy, M. Fourment, S. Mardy, S. Ly, S. Vong); Ministry of Agriculture, Fisheries and Forestry, Phnom Penh (S. Sorn, D. Holl); Institut Pasteur, Paris, France (V. Enouf, S. van der Werf); Hong Kong University, Hong Kong Special Administrative Region, People’s Republic of China (J.S.M. Peiris)

**Keywords:** H5N1, epidemiology, transmission, poultry, wild birds, Cambodia, Vietnam, Thailand, viruses, influenza, dispatch

## Abstract

To determine the origin of influenza A virus (H5N1) epizootics in Cambodia, we used maximum-likelihood and Bayesian methods to analyze the genetic sequences of subtype H5N1 strains from Cambodia and neighboring areas. Poultry movements, rather than repeated reintroduction of subtype H5N1 viruses by wild birds, appear to explain virus circulation and perpetuation.

From 2004 through 2007, a total of 26 outbreaks of influenza A virus (H5N1) infection have occurred in poultry in Cambodia, and 7 human cases have been reported. Subtype H5N1 infections were observed primarily during the dry season, from January through April. The origin of these epizootics in Cambodia remains unclear. Here we have used maximum likelihood and Bayesian methods to analyze the Cambodian virus genetic sequences, together with those obtained from other H5N1 viruses isolated in neighboring countries since 2004, to understand that patterns of virus transmission.

## The Study

We analyzed the sequences of all 8 genomic segments of 33 subtype H5N1 viruses sampled in Cambodia from 2004 through 2007, together with publicly available sequences from 116 isolates from Southeast Asia obtained from GenBank. We included all subtype H5N1 viruses sequences from southern Vietnam. In Cambodia, we sequenced systematically at least 2 strains from each infected flock and obtained a total of 137 sequences. When sequences obtained from viruses isolated during the same outbreak site were identical, we used only one for the analyses. After alignment, phylogenetic analyses were conducted by using maximum-likelihood and Bayesian methods (*1*,*2*).

**Figure 1:**
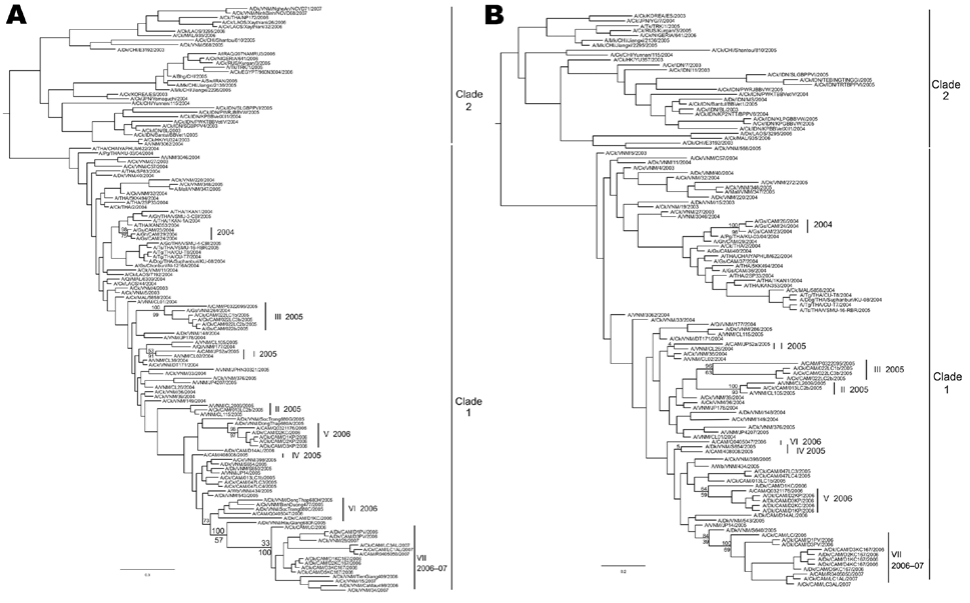
Phylogenetic relationships of the hemagluttinin (HA) (A) and neuraminidase (NA) (B) genes of 33 Cambodian strains and of representative influenza A viruses (H5N1). Trees were generated by Bayesian analysis using MrBayes v3.1 software (*2*). Numbers above and below branches indicate Bayesian posterior probability and maximum likelihood bootstrap values (PHYML v2.4 software, www.atgc-montpellier.fr/phyml), respectively. Analysis was based on nucleotides 28–1578 of the HA gene and 67 to 1248 of the NA gene. Both trees were rooted to A/goose/Guangdong/6/96. Scale bar indicates 0.3 and 0.2 substitutions per site for HA and NA genes, respectively. Dk, duck; Ck, chicken; Gs, goose; Bhg, bar headed goose; Pg, pigeon; Gh, gray heron; Ql, quail; Mall, Mallard duck; Tg, tiger; CAM, Cambodia, VNM, Vietnam, THA, Thailand, MAL, Malaysia, CHI, People’s Republic of China; TRK, Turkey; RUS, Russia, JPN, Japan. Cambodian subtype H5N1 viruses that are grouped in closely related phylogenetic sublineages and are denoted by sublineage number (arbitrarily numbered I–VII for purposes of molecular epidemiology) and these sublineages are also denoted in the map (Figure 2) to show where these viruses have found and the likely virus introduction routes. It is to be emphasized that all these viruses are within the World Health Organization (WHO) clade 1, and these sublineage numbers are not to be confused with the WHO clade/subclade nomenclature.

**Figure 2:**
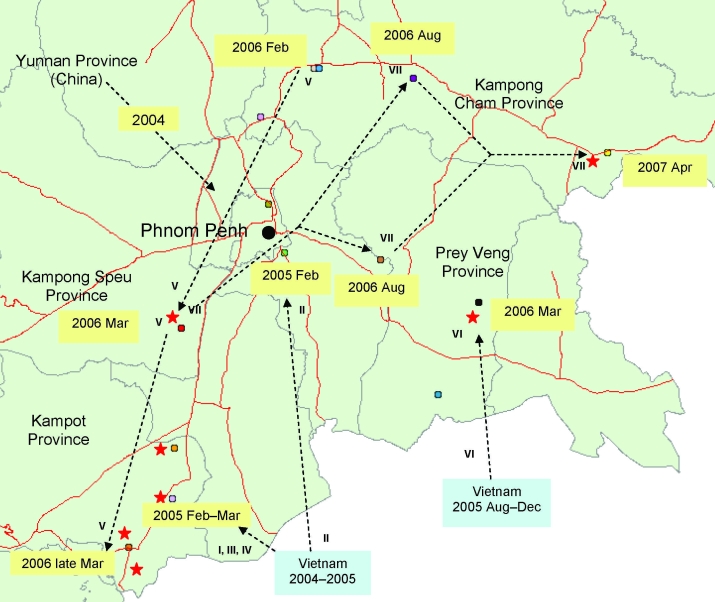
Map of Cambodia showing the locations of influenza A (H5N1) outbreaks in poultry (circles) and human cases (stars) detected since 2005. Arrows are proposed to illustrate the hypothetical paths of introduction of H5N1 virus sublineages (see Figure 1) in Cambodia from its neighboring countries. A sublineage number adjacent to the arrow implies that the respective sublineage viruses are found at the start and the end of the arrow with the years of the detection noted (Appendix Table). Molecular characteristics of hemagluttinin (HA) sequence and hemagglutination inhibition (HI) tests of Cambodian viruses and some reference subtype H5N1 strains. A) H5 aa numbering. RBS, receptor binding site; DEL, deletion. B) BHG, bar-headed goose; VNM, Vietnam; CAM, Cambodia; INDO, Indonesia. Numbers I–VII refer to sublineages; within sublineage VII, PV06: group of viruses represented by the strain A/Duck/Cambodia/D1PV/2006; KC06: group of viruses represented by the strain A/Duck/Cambodia/D1KC1672006; CAM07: group of viruses represented by the strain A/Cambodia/R0405050/07; VNM06–07: group of viruses isolated in South Vietnam in 2006 and 2007. C) Antigenic characterization was performed by using the HI assay with ferret antisera raised to World Health Organization reference subtype H5N1 viruses. Numbers are the results of the differences between the log_2_(HI titer/10) of the reference virus and the virus tested. D) For HI tests, the virus A/BHG/Qinghai Lake/1A/2005 was tested against this homologous serum, while ferret serum against A/Turkey/15/2005 was tested against the reassortant virus NIBRG-23 derived from A/Turkey/15/2005. A/BHG/Qinghai Lake/1A/2005 and A/Turkey/15/05 are both clade 2.2 viruses.

All H5N1 viruses that were detected in Cambodia from 2004 through 2007 belong exclusively to clade 1, genotype Z (Figure 1, panel A) (*3*,*4*). The hemagluttinin (HA) phylogenetic tree shows that viruses isolated from Yunnan in 2002 and 2003 are at the origin of all clade 1 viruses from Thailand, Cambodia, and Vietnam (Figure 1, panel A). Sequences obtained from viruses first isolated in Cambodia in January 2004 cluster with a large number of sequences of H5N1 viruses isolated in 2004 and 2005 in Thailand (Figure 1). Subsequent Cambodian subtype H5N1 isolates are grouped into closely related phylogenetic sublineages and are denoted by sublineage number (arbitrarily numbered I–VII for purposes of molecular epidemiology), and these sublineages are also denoted in the map (Figure 2) to show their geographic locations and relationships to likely virus introduction routes. All of these viruses are within the World Health Organization (WHO) clade 1, and these sublineage numbers are not to be confused with the WHO clade/subclade nomenclature.

In 2005, all 4 reported human cases were identified in a limited area of Kampot Province, near the Vietnamese border (Figure 2). Sequences of A/Cambodia/JP52a/2005 detected in January 2005 cluster with strains isolated in 2004 in Southeast Vietnam. The HA gene sequences fall within a cluster of viruses, closely related phylogenetically within clade 1, which we arbitrarily named sublineage I (Figure 1, panel A; Figure 2). Similarly, the strains A/chicken/Cambodia/013LC2b/2005, A/Cambodia/P0322095/2005, and A/Cambodia/408008/2005 detected during March–April 2005 in Kampot and Kandal provinces form separates clusters with sequences of South Vietnamese strains isolated in 2004–2005, namedand named sublineages II, III, and IV, respectively (Figures 1 and 2).

When all the major outbreak sites were plotted on a map of Cambodia, especially sites where viruses from sublineage V (Figure 1, panel A; Figure 2) were isolated in February and early and late March 2006, respectively, it appears that subtype H5N1 virus spread north to south following a main road (Figure 2). Virus A/Cambodia/Q0405047/2006 detected in March 2006 (sublineage VI) is phylogenetically closely related to viruses detected in 2 Vietnamese provinces between August and December 2005 (Figure 1, panel A). We observed 2 virus strains, A/duck/Cambodia/D2KC/2006 and A/duck/Cambodia/D1KC/2006, which were isolated in poultry at the same time and location (February 2006 in Kampong Cham Province), but belong to different sublineages, V and VI, respectively. The HA sequences of the 2 viruses differ by 2 nonsynonymous substitutions that lead to amino acid changes at positions 184 and 195, and by 23 synonymous substitutions. The neuraminidase (NA) gene sequences of both viruses are phylogenetically closely related to the same NA sublineage V, which suggests that a reassortment event occurred (Figure 1, panel B).

Sublineage VII contains 5 separate branches (Figure 1, panel A), which correspond viruses isolated from different geographic locations at different times: Kampong Speu in March 2006 (LC/2006), Kampong Cham in August 2006 (e.g., D1KC167/2006), Prey Veng in August 2006 (e.g., D1PV/2006, which also clusters with a 2007 Vietnamese virus), Kampong Cham in April 2007 (e.g., R0405050/2007) and Vietnam 2006/2007 (Figure 1, panel A). A/Chicken/Vietnam/29/2007 is closely related phylogenetically to Cambodian viruses detected in August 2006 and not closely related to the group of Vietnamese strains from 2006/2007. This suggests that the 2007 Vietnamese strains also have various parental origins, probably related to geographically distinct circulation areas.

For all gene segments, the percentage of homology varied from 98% (for the HA segment) to 99.8% (for the M1 coding gene) between sequences of the first and the latest virus for which complete genome sequences were obtained. Mutations S123P and S129A, R to G substitution in the HA cleavage site, and S155N and K189N mutations at antigenic site B (which could explain the antigenic drift measured) are characteristics that seem to have become established in the latest Cambodian isolates (Appendix Table).

## Conclusions

Cambodia is essentially a poultry-importing country. The first poultry deaths were observed in semi-industrial chicken farms that imported broiler and layer parental stocks from a sister company in Thailand, where outbreaks occurred contemporaneously. Whether the viruses were introduced from Vietnam into Cambodia or vice versa cannot be ascertained from the phylogenetic evidence. However, given the known direction of poultry imports from Vietnam to Cambodia, multiple introductions of subtype H5N1 viruses most likely occurred through illegal trading in poultry from Vietnam; this hypothesis would explain how several sublineages emerged in Cambodia in 2005 and 2006. The national veterinary surveillance system in Cambodia is probably not sufficiently sensitive to detect outbreaks resulting from a low prevalence of circulating subtype H5N1. The silent circulation of subtype H5N1 virus in vaccinated poultry (e.g., in ducks) in Vietnam in October 2005 could explain why Vietnam appeared free of subtype H5N1 infections from December 2005 through October 2006, while viruses were still able to cross the border and to infect nonimmune (nonvaccinated) Cambodian poultry (*5*). Similar to the situation in Vietnam (*6*), Cambodian farmers tend to release ducks in the paddy fields, particularly after harvesting periods in which duck flocks could feed on the harvest’s leftovers. Seasonality could then be explained by higher duck density (*7*,*8*). Viruses in Cambodia very likely represent multiple external introductions rather than becoming established within Cambodia on a continuous basis. This may be related to the fact that the poultry density in Cambodia is lower (average 94 heads/km^2^) (*9*) compared with that in neighboring countries such as Thailand and Vietnam (average 640 heads/km^2^) (*10*), and one may speculate that a minimum density of poultry is required for maintaining an endemic virus.

Lake Tonle Sap (16,000 km^2^ surface) and other lakes and wetlands host numerous wild birds, mostly resident or regional migratory birds (M. Gilbert, pers.comm.), which could be involved in the spread of subtype H5N1 virus. Should this mode of transmission play a role, it would probably be limited to local or regional transmission, especially since viruses from other clades (i.e., clade 2.2) that are imported to neighboring countries by migratory birds from the People’s Republic of China, have never been detected in Cambodia.

Sublineage VII–specific mutations (i.e., R325G, S123P) in strains from Cambodia and Vietnam suggest that this new sublineage became endemic in the southern Cambodia–Mekong Delta region during 2006. The phenomenon of virus spread through poultry trading, particularly along the roads, accounts for maintenance and extension of virus within a given region and sustains the risks of transmission to humans.

## Supplementary Material

Appendix Table Molecular characteristics of HA sequence and HI tests of influenza virus A (H5N1) isolates from Cambodia, 2004-2007, and reference subtype H5N1 strains*
