# Supplementary material for: Molecular Epidemiology of Clade 1 Influenza A Viruses (H5N1), Southern Indochina Peninsula, 2004–2007
Source: Emerg Infect Dis. 2009 Oct;15(10):1641–4. doi: 10.3201/eid1510.090115 (PMC2866389; doi:10.3201/eid1510.090115)
Supplement: Appendix Table — Molecular characteristics of HA sequence and HI tests of influenza virus A (H5N1) isolates from Cambodia, 2004-2007, and reference subtype H5N1 strains* [file 09-0115_appT-s1.pdf]

Appendix Table. Molecular characteristics of HA sequence and HI tests of influenza virus A (H5N1) isolates from Cambodia, 2004–2007, and reference subtype H5N1 strains*

	HA regions and amino acids†																Ferret serum§			
Reference virus, sublineages‡	22	Site E 86	94	123	Site B 124	RBS 133	RBS 136	Site A 138	Site A 140	Site A 141	Site B 155	175	184	Site B 189	195	210	Cleavage site 325	A/VNM/ 1194/2004	A/INDO/ 05/2005	A/Turkey/15/05– A/BHG/Qinghai Lake/1A/2005¶	A/Anhui/ 1/2005
VNM/1194/2004	K	V	D	S	S	S	P	Q	K	S	S	L	A	K	T	V	R	X	4	2	4.5
A/INDO/05/2005	K	T	S	S	D	S	P	L	S	S	S	L	A	R	T	V	S	1.5	X	2	4.5
A/BHG/Qinghai lake/1A/2005¶	K	A	N	S	D	S	P	Q	T	T	N	L	A	R	T	V	T	2	2	X	4.5
A/Anhui/1/2005	K	A	N	S	D	S	P	Q	T	T	N	L	A	K	T	V	R	1.5	0.5	2	X
CAM 2004	K	V	D	S	S	S	P	Q	K	S	S	L	A	K	T	V	R	0	2.5	1.5	4.5
I	K	V	D	S	S	S	L	Q	Q	P	S	M	A	K	T	V	R	1.5	7	3	5
II	K	V	N	S	S	A	P	Q	K	S	S	M	A	K	T	V	R	1	3.5	1.5	4.5
III	K	V	D	S	S	S	P	L/M	K	S	S	M	A	K	T	V	R	0	3.5	1.5	4.5
IV	R	V	D	S	N	A	P	Q	K	S	S	M	A	K	T	V	R	–2	2.5	2	3
V	K	V	D	S	S	A	P	Q	K	S	S	M	E	K	I/T	V	Del/R	1	3.5	2	4.5
VI	K	V	D	S	S	A	P	Q	K	S	S	M	A	K	T	V	R	–2.5	3	3	5
VII																					
PV06	K	V	D	P	S	A	P	Q	K	S	S	M	A	K	T	T	G				
KC06	K	V	D	P	N	A	P	Q	K	S	S	M	A	K	T	T	R	1			
CAM07	K	V	D	P	S	A	P	Q	K	S	N	M	A	N	T	T	G	2–4	7	3	5
VNM06-07	K	V	V	P	S	A	P	Q	K	S	S	M	A	K	T	V	G				

*HA, hemagglutinin; HI, hemagglutination inhibition; RBS, receptor binding site; Del, deletion; BHG, bar-headed goose; VNM, Vietnam; CAM, Cambodia; INDO, Indonesea; WHO, World Health Organization.

†H5 animo acids numbering.

‡Numbers I–VII refer to sublineages; within sublineage VII, PV06 = group of viruses represented by the strain A/duck/Cambodia/D1PV/2006; KC06 = group of viruses represented by the strain A/duck/Cambodia/D1KC167/2006; CAM07 = group of viruses represented by the strain A/Cambodia/R045050/2007; VNM06-07 = group of viruses isolated in South Vietnam in 2006 and 2007.

§Antigenic characterization was performed using the HI assay with ferret antisera raised to WHO reference subtype H5N1 viruses. Numbers are the results of the differences between the $\log_2$ (HI title/10) of the reference virus and the virus tested.

¶For HI tests, the virus A/BHG/Quinghai Lake/1A/2005 was tested against the reassortant virus NIBRG-23 derived from A/Turkey/15/2005. A/BHG/Quinghai Lake/1A/2005, and A/Turkey/15/2005 are both clade 2.2 viruses.
